# Changing Epidemiology of Acute Viral Respiratory Infections in Hospitalized Children: The Post-Lockdown Effect

**DOI:** 10.3390/children9081242

**Published:** 2022-08-17

**Authors:** Marco Maglione, Antonia Pascarella, Chiara Botti, Giuseppe Ricci, Fiorella Morelli, Fabiana Camelia, Alberto Micillo, Camilla Calì, Fabio Savoia, Vincenzo Tipo, Antonietta Giannattasio

**Affiliations:** 1 Pediatric Emergency Unit, Santobono-Pausilipon Children’s Hospital, 80129 Naples, Italy; 2 Laboratory of Clinical Pathology, Santobono-Pausilipon Children’s Hospital, 80129 Naples, Italy; 3 Childhood Cancer Registry of Campania, Santobono-Pausilipon Children’s Hospital, 80129 Naples, Italy

**Keywords:** respiratory syncytial virus, children, acute respiratory infections, COVID-19

## Abstract

Several reports highlighted how public health measures aimed at limiting severe acute respiratory syndrome Coronavirus-2 (SARS-CoV-2) circulation have likely contributed to reducing the circulation of other respiratory viruses, particularly during the first year of the COVID-19 pandemic. We evaluated the epidemiology of acute respiratory infections in a large cohort of hospitalized children during the third year of the pandemic (2021–2022). We retrospectively analyzed data from the health records of children (<14 years) hospitalized for acute respiratory infections between 1 July 2021 and 31 March 2022. A total of 1763 respiratory panels were collected. Overall, 1269 (72%) panels hadpositive results for at least one pathogen. Most positive panels (53.8%) belonged to patients aged 1–12 months. The most detected pathogen was respiratory syncytial virus (RSV) (57.8% of positive panels). The RSV peak occurred in November 2021. Nine hundred and forty-five (74.5%) panels were positive for one pathogen while three hundred and twenty-four (25.5%) showed multiple infections. Patients with multiple infections were significantly older than those with a single infection. The 2021–2022 peak of RSV infection in Italy occurred earlier than in the previous pre-pandemic seasons. A high number of children have been hospitalized because of acute viral infections also due to less aggressive viruses.

## 1. Introduction

Acute respiratory infections (ARIs) are the leading infectious disease among children worldwide and are responsible for a substantial proportion of pediatric hospitalizations and deaths [1]. Globally, the higher morbidity and mortality for viral lower respiratory infections have been reported in association with respiratory syncytial virus (RSV) [2]. Despite the relevant burden of pediatric viral ARIs, the development of effective preventive or treatment strategies still represents a challenge. A recent Cochrane systematic review aimed to assess the effectiveness of physical interventions (e.g., use of face masks and hand hygiene) to reduce the spread of acute respiratory viruses other than severe acute respiratory syndrome Coronavirus-2 (SARS-CoV-2) and concluded that the low compliance with the interventions during the studies hampers drawing firm conclusions and generalizing the findings [3]. Nevertheless, several reports highlighted how public health measures aimed at limiting SARS-CoV-2 circulation have likely contributed to reducing the circulation of other respiratory viruses, particularly during the first year of the COVID-19 pandemic, when severe restriction measures had been applied [4,5,6,7].

In this study, we retrospectively evaluated the epidemiology of ARIs in a large cohort of hospitalized children during the third year of the COVID-19 pandemic.

## 2. Materials and Methods

Data from the health records of pediatric patients (<14 years) hospitalized for ARIs between 1 July 2021 and 31 March 2022 were retrospectively analyzed. Patients with a clinical diagnosis of ARI who did not undergo a respiratory panel were excluded from the analysis.

According to the World Health Organization (WHO), ARI isdefined as an acute respiratory illness with a history of fever or measured fever of ≥38 °C and cough, with onset within the past 10 days, requiring hospitalization [8]. Nevertheless, as the presence of fever is not required for the diagnosis of bronchiolitis, we also included patients without fever, but with cough, nasal obstruction, nasal flaring, tachypnea, and/or hypoxia, fulfilling the clinical definition of bronchiolitis [9].

Respiratory pathogens were searched by FilmArray Respiratory Panel (FilmArray^®^ Respiratory Panel BioFire Diagnostics LLC 390; Wakara Way, Salt Lake City, UT, USA) on naso-pharyngeal swabs. This test identifies Influenza A virus (FluA) (H1N1, H1N1 2009, and H3N2), influenza B virus (FluB), RSV, human parainfluenza viruses 1–4 (PIVs 1–4), adenovirus (ADV), rhino/enteroviruses (HRV/EV), metapneumovirus (HMPV), human coronaviruses (HCoVs) (229E, HKU1, OC43, and NL63), Middle East Respiratory Syndrome virus (MERS), SARS-CoV-2, Bordetella pertussis (BORp), Bordetella parapertussis (BORpa), Chlamydia pneumoniae (CLAMP), and Mycoplasma pneumoniae (MYCOP). Positive samples for SARS-CoV-2 were also tested for quantitative detection of SARS-CoV-2 nucleic acids.

Patients’ clinical information (gender, age, season of hospital admission), and microbiological information (detected viruses, single infection versus co-infection) were considered for the analysis.

### Statistical Analysis

A statistical analysis was performed by two experienced statisticians. Data were reported as number and percentages. Wilcoxon’s rank sum test was used to evaluate continuous variables. Multivariate and univariate logistic regression models were developed to assess independent variables associated with the presence/absence of multiple infections. The independent variables examined were gender, age, and season of hospital admission.

## 3. Results

We analyzed 1763 respiratory panels. Overall, 1269 (72%) panels resulted in a positive response for at least one pathogen. The age distribution of positive panels was: 131 out of 210 (62.4%) performed panels in newborns; 683/947 (72.1%) in patients aged 1–12 months; 177/226 (78.3%) in patients aged 12–24 months; 219/266 (82.3%) in the group 2–5 years; and 59/114 (51.8%) in the group over 5 years.

**Figure 1:**
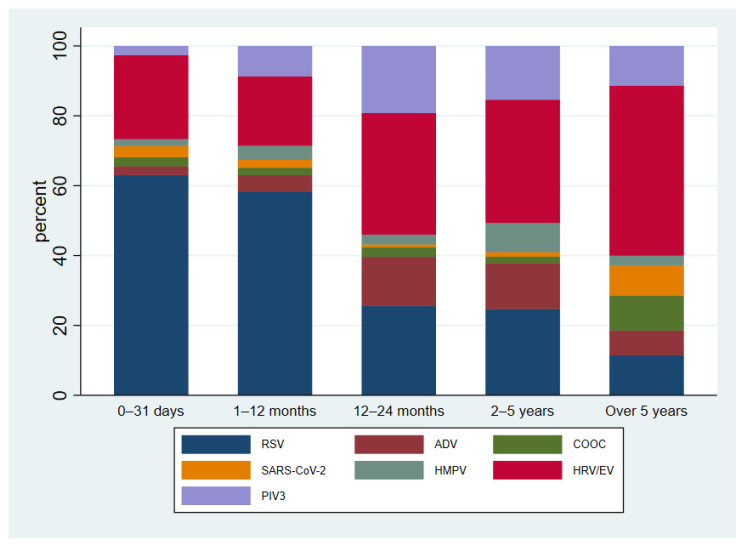
Distribution of detected pathogens according to patients’ age.

Overall, most panels (947/1763, 53.8%) were performed in children aged 1–12 months. The distribution of specific pathogens according to patients’ age is reported in Figure 1. No significant seasonal difference was observed in the proportion of positive panels over the total performed. Nevertheless, in Autumn, the number of processed panels (*n* = 1124) was significantly higher than Winter (*n* = 332) and Summer (*n* = 307).

**Figure 2:**
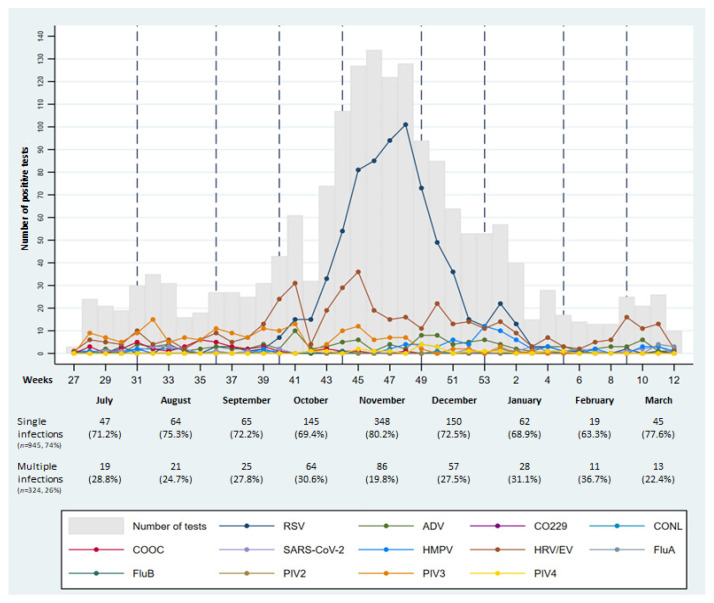
Pathogen distribution according to weeks of observation (from 1 July 2021 to 31 March 2022) and distribution of single versus multiple infections according to months of observation.

**Figure 3:**
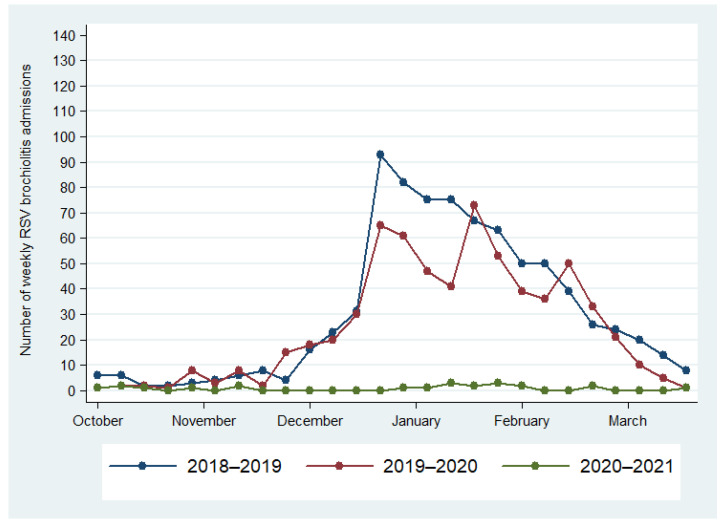
Hospitalization due to RSV infection during the pre-pandemic seasons (2018–2019 and 2019–2020) and during the 2020–2021 year of SARS-CoV-2 pandemic.

The most commonly detected pathogen was RSV. In total, 945 (74.5%) panels were positive for one pathogen, while 324 (25.5%) showed multiple infections: 2 pathogens in 255 (78.7%) cases, 3 pathogens in 58 (17.9%), 4 pathogens in 10 (3.1%), and 5 pathogens in 1 case. The most common pathogen in multiple infections was HRV/EV (56.8% of total HRV/EV) followed by RSV (23.3% of total RSV) (Table 1). Patients with multiple infections were significantly older than those with a single infection (20.5 ± 26.6 versus 15.35 ± 27.2 months, *p* < 0.001). Particularly, the univariate and multivariate analysis showed an increased risk of multiple infections for the age groups 12–24 months and 2–5 years, in comparison with younger children (Table 2). The distribution of respiratory pathogens differed according to the observation period, whereas the distribution of co-infections versus single infection did not significantly differ according to the month of the patient’s admission (Figure 2). It is useful to note that the peak of RSV was observed in November 2021 and it lasted 11 weeks. Compared to historical data from our hospital, no outbreaks of RSV were reported in 2020, while previous outbreaks (2018–2019 and 2019–2020) occurred between the end of December and the beginning of January (Figure 3). As expected, the majority (80%) of patients with RSV infection were under 12 months of age (Figure 1). Namely, patients with a single RSV infection had a mean age of 9.3 ± 18.5 months, whereas the mean age of patients with multiple infections including RSV was 15.7 ± 24.8 months. Patients with an RSV infection older than 12 months were aged 12–24 months and 2–5 years in equal proportions.

## 4. Discussion

In the present study, we enrolled all children hospitalized because of ARI of documented etiology in the post-lockdown period. Our data show that the epidemiology of ARIs in the 2021–2022 season has changed, with an unexpected peak of RSV in November 2021, significantly earlier than the usual peak, that in Italy generally occurs one to three months later [10].

During the first year of the COVID-19 pandemic, the circulation of RSV stopped immediately as a consequence of the measures adopted to control SARS-CoV-2 circulation [4,5,6,7,11]. Since then, RSV circulation has been observed only in a few countries during the 2020/21 Winter, with epidemics starting several weeks later than usual and presenting a shorter duration and decreased peak size compared to the previous seasons [11]. Furthermore, at our large Pediatric Hospital, we observed a drastic decrease in the number of hospital admissions due to RSV during the first year and a half of the pandemic.

Most published studies stopped the analysis afterthe first months of 2021 [6,11,12,13,14] and were thus unable to predict the magnitude or length of the RSV surge that we described in our study. The first European report of bronchiolitis epidemiology after lockdowns has been recently published [10]. The authors reported a bronchiolitis season starting earlier than usual in the 2021–2022 season in Italy. Nevertheless, despite its multicenter design, this study only included patients with a clinical diagnosis of bronchiolitis, and the microbiological analysis was limited to 264 children, representing about one third of the enrolled subjects. Furthermore, the study period was limited to July 2021–January 2022, and data regarding the following months, often interested by the RSV peak, were not included [10].

The present study confirms and extends these findings. Indeed, as our center represents the largest tertiary care Pediatric Hospital in Southern Italy, our data derive from a huge sample of patients, with more than 1700 respiratory panels analyzed over nine months. Therefore, our analysis provides a wide and robust picture of the circulation of respiratory viruses among children in the post-lockdown period. The RSV peak that we documented in November 2021 occurred earlier than previous seasons and, in line with other European reports [11], it lasted only 11 weeks compared with the 14–15 weekduration of pre-pandemic years.

Data from other countries reported an increased median age of children with RSV infection compared to previous seasons: 4.8 months in 2020/21 versus 2.2 to 3.1 months in the seasons 2016/17–2019/20 [11]. Although RSV mostly affected patients aged <1 year, in our study the mean age of RSV-infected patients was higher than that reported in pre-pandemic cohorts. Furthermore, a relevant proportion (about 20%) of older children hospitalized for ARI presented RSV positivity as an isolated agent. This increased age suggests an expansion of the cohort of RSV-naïve patients due to the pandemic’s restrictive measures.

Interestingly, an additional finding from our analysis was the observation of two peaks in HRV/EV circulation that were slightly anticipated in comparison to the RSV peak (early October and early November). Furthermore, a nadir in HRV/EV circulation coincided with the highest number of detected RSV infections. Unlike RSV, HRV, which was the second most commonly isolated pathogen in our cohort, does not show a typical seasonality [15], and a clear epidemiological change is therefore difficult to document. Nevertheless, our findings are in line with the results from a study exploring RSV-HRV co-detection in infants [16]. The authors described a negative association between these two pathogens and hypothesized a viral interference entailing that the concurrent or prior presence of one virus results in a measurable reduction in the presence of another virus. Such a mechanism of viral interaction might be based on a biological phenomenon, possibly determined by an interferon response induced during RSV infection that may mediate an inhibition of HRV, decreasing its infectious potential [16].

In the Campania region (Southern Italy), measures to control the spread of SARS-CoV-2 were longer and stricter compared to other Italian regions and to other European countries [7,11,17,18]. The lockdown determined the closure of all grade schools and the restriction of children’s group activities from5 March to October 2020. In the following months (between the end of 2020 and the beginning of 2021), schools remained closed again for a long period. Therefore, during the first two years of the pandemic, we had very few cases of children hospitalized because of RSV [19]. Since July 2021, with the decreased incidence of SARS-CoV-2 infection, the diffusion of the anti-SARS-CoV-2 vaccination program, and the milder course of COVID-19, most restrictions were progressively reduced, with children regularly attending school and other social activities since September 2021. Consequently, we experienced a high spread of infectious diseases, mainly affecting the respiratory tract, and a changed seasonality of RSV incidence compared to the usual pre-pandemic RSV season [20].

Compared to the available literature, our research has several strengths. First, all analyzed patients had received a diagnosis of ARI of documented etiology. Secondly, we provided a complete analysis not limited to RSV, but also including a wide range of detectable respiratory pathogens. Finally, the study period was prolonged to March 2022, thus covering all the months usually involved by a significant spread of respiratory infections. This longer study period confirmed the progressive disappearance of RSV during January and February 2022, i.e., the months usually characterized by its peak in the pre-COVID-19 seasons. On the other hand, the retrospective design, and the lack of correlation between detected pathogens and clinical severity are among the main drawbacks of our study. Moreover, as we analyzed data up to the 12th week of 2022, we cannot exclude further changes in the proportions of circulating respiratory viruses in the following months. Finally, only data of RSV admission during the pre-pandemic period were available for analysis in our study; therefore, we could not compare the 2021–2022 season with pre-pandemic data for viruses other than RSV.

## 5. Conclusions

The present study documents a substantial outbreak of pediatric ARIs from several viral agents as a likely effect of the progressive discontinuation of public health measures established during the first years of the COVID-19 pandemic to limit SARS-CoV-2 circulation. Particularly, an unexpected RSV seasonality was observed in 2021–2022, with an earlier peak of shorter duration in comparison to previous seasons. Among the other respiratory pathogens detected, HRV has been responsible for significant morbidity both as a single agent and in multiple infections. Monitoring of the circulation of respiratory pathogens represents a valuable tool for a better understanding of the cloudy dynamics of viral seasonality and of the impact of public health measures on periodic outbreaks. This could also provide useful information for preventive strategies and planning of health resources.

## Figures and Tables

**Table 1:** Distribution of respiratory pathogens as total infections and their role in case of multiple infections.

	Total Infections * (*n* = 1674)		Multiple Infections (*n* = 324)	
	*n*	%	*n*	%
**RSV**	733	43.8	171	52.8
**ADV**	124	7.4	98	30.2
**CO229E**	4	0.2	2	0.6
**CONL**	17	1.0	10	3.1
**COOC**	42	2.5	22	6.8
**SARS-CoV-2**	36	2.2	15	4.6
**HMPV**	72	4.3	42	13
**HRV/EV**	433	25.9	246	75.9
**FluA**	11	0.7	5	1.5
**FluB**	1	0.1	1	0.3
**PIV2**	2	0.1	2	0.6
**PIV3**	181	10.8	100	30.9
**PIV4**	18	1.1	15	4.6

* Number of total infections deriving from the sum of all detections of each pathogen both in single and multiple infections. RSV, Respiratory Syncytial Virus; ADV, adenovirus; CO229, coronavirus 229E; CONL, coronavirus NL63; COOC, coronavirus OC43; SARS-CoV-2, Severe Acute Respiratory Syndrome Coronavirus 2; HMPV, metapneumovirus; HRV/EV, rhino/enterovirus; FluA, Influenza A virus; FluB, Influenza B virus; PIV2, parainfluenza virus 2; PIV3, parainfluenza virus 3; PIV4, parainfluenza virus 4. Despite being searched for, Coronavirus HKU1, Middle East Respiratory Syndrome virus, parainfluenza virus 1, Bordetella pertussis, Bordetella parapertussis, Chlamydia pneumoniae, and Mycoplasma pneumoniae are not reported as they were detected in no samples.

**Table 2:** Multivariable and univariate logistic regression models for independent variables associated with presence/absence of multiple infections.

	Multiple Infections				Univariate Analysis				Multivariate Analysis			
	Yes (*n* = 324, 26%)		No (*n* = 945, 74%)									
	*n*	%	*n*	%	OR	95% CI		*p*	OR	95% CI		*p*
**Gender**					0.89	0.69	1.14	0.36	0.81	0.63	1.06	0.12
Male	181	26.6	500	73.4								
Female	143	24.3	445	75.7								
**Age group**												
0–31 days	24	18.3	107	81.7								
1–12 months	138	20.2	545	79.8	1.13	0.70	1.83	0.62	1.11	0.69	1.80	0.67
12–24 months	65	36.7	112	63.3	2.59	1.51	4.43	<0.001	2.61	1.51	4.49	<0.001
2–5 years	82	37.4	137	62.6	2.67	1.59	4.49	<0.001	2.72	1.61	4.59	<0.001
Over 5 years	15	25.4	44	74.6	1.52	0.73	3.17	0.26	1.43	0.68	3.02	0.34
**Season**												
Summer	60	27.5	158	72.5								
Autumn	204	24.2	638	75.7	0.84	0.60	1.18	0.32	1.05	0.74	1.49	0.79
Winter	60	28.7	149	71.3	1.06	0.70	1.62	0.79	1.21	0.79	1.87	0.38

## Data Availability

Not applicable.
